# Perception and discrimination of real-life emotional vocalizations in early blind individuals

**DOI:** 10.3389/fpsyg.2024.1386676

**Published:** 2024-05-09

**Authors:** Chiara Ferrari, Maria Arioli, Doron Atias, Lotfi B. Merabet, Zaira Cattaneo

**Affiliations:** ^1^Department of Humanities, University of Pavia, Pavia, Italy; ^2^IRCCS Mondino Foundation, Pavia, Italy; ^3^Department of Human and Social Sciences, University of Bergamo, Bergamo, Italy; ^4^Department of Psychology, Hebrew University of Jerusalem, Jerusalem, Israel; ^5^The Laboratory for Visual Neuroplasticity, Department of Ophthalmology, Massachusetts Eye and Ear, Harvard Medical School, Boston, MA, United States

**Keywords:** intense emotions, real-life vocalizations, blindness, compensatory mechanisms, arousal

## Abstract

**Introduction:**

The capacity to understand others’ emotions and react accordingly is a key social ability. However, it may be compromised in case of a profound sensory loss that limits the contribution of available contextual cues (e.g., facial expression, gestures, body posture) to interpret emotions expressed by others. In this study, we specifically investigated whether early blindness affects the capacity to interpret emotional vocalizations, whose valence may be difficult to recognize without a meaningful context.

**Methods:**

We asked a group of early blind (N = 22) and sighted controls (N = 22) to evaluate the valence and the intensity of spontaneous fearful and joyful non-verbal vocalizations.

**Results:**

Our data showed that emotional vocalizations presented alone (i.e., with no contextual information) are similarly ambiguous for blind and sighted individuals but are perceived as more intense by the former possibly reflecting their higher saliency when visual experience is unavailable.

**Disussion:**

Our study contributes to a better understanding of how sensory experience shapes ememotion recognition.

## Introduction

To successfully interact in social contexts we need to react to communication signals, including emotional signals expressed by others. Observers can perceive emotions from others’ facial, bodily, vocal, and verbal expressions, and this perception is further informed by contextual information. Among the available emotional cues, non-verbal vocalizations represent powerful primitive signals (e.g., Cowen et al., 2019) that prompt reactions (e.g., Warren et al., 2006), but whose valence may not be so obvious when no other contextual information is available (Atias et al., 2019). Indeed, Atias et al. (2019) showed that participants could not distinguish the valence of human non-verbal vocalizations occurring in real intense positive (e.g., reacting to the reunion with a loved one) and negative (e.g., reacting to an attacker invading one’s home) situations when these vocalizations were presented alone. However, when the affective vocalizations were matched with the corresponding visual contexts, participants immediately understood their valence correctly. As another example, Lavan et al. (2015) demonstrated that facial expressions drive the emotional interpretation of intense laughter and crying, further supporting the importance of information drawn from visual contexts.

Although we are usually presented with “contextualized” vocalizations, this may not be the case of a blind person who cannot rely on visual cues such as facial expressions or gestures in interpreting vocal emotional signals. Consistent experimental evidence suggests that blind people compensate for the lack of visual input and experience by increased sensitivity of their tactile (e.g., Cattaneo et al., 2010a; Bauer et al., 2015; Gurtubay-Antolin and Rodríguez-Fornells, 2017) and auditory senses (for a recent review, Sabourin et al., 2022). For instance, in the auditory domain blind individuals outperform sighted controls in the processing of pitch changes (e.g., Arnaud et al., 2018), localization (e.g., Battal et al., 2020), voice recognition (Hölig et al., 2014, 2015), as well as in auditorily presented sentence comprehension (Loiotile et al., 2020). Available findings on the processing of vocal emotions in blind individuals are mixed, with some studies reporting enhanced discrimination of emotional vocalizations in blind participants compared to the sighted (Klinge et al., 2010), others reporting no differences (Gamond et al., 2017) or even worse performance in the blind (e.g., Martins et al., 2019; Chen and Whitney, 2022; Sarzedas et al., 2024). Interestingly, blindness has also been associated with physiological differences in the way emotional vocalizations are processed: Klinge et al. (2010) reported increased amygdala activation to negatively valenced stimuli in early blind participants [see Klinge et al. (2012)], suggesting that emotional vocalizations may be perceived as more salient when visual input is lacking. Furthermore, recent electrophysiological evidence suggests that early blindness relates to facilitated brain processing of authenticity of vocal emotional expressions (Sarzedas et al., 2024) and prior evidence showed ERP attention effects in blind individuals across a broader range of different emotional prosodies compared to sighted individuals (Topalidis et al., 2020).

To shed light on whether a lack of visual input and experience is associated with an improved capacity to interpret emotional vocalizations, we presented the same intense emotional vocalizations [as previously used by Atias et al. (2019)] to a group of early blind individuals and a group of normally sighted controls. Participants were asked to evaluate the stimuli as emotionally positive or negative, as well as to rate their intensity.

## Methods

### Participants

Twenty-two early blind individuals (seven females), mean age 43.3 years ±13.2 SD (age range 20–61 years), mean education level of 16 ± 3.92 years, and 22 normally sighted participants (seven females), mean age 43.0 ± 13.6 years (age range 23–61 years), mean education level of 16.2 ± 3.39 years, took part in the experiment. The two groups did not differ in terms of age, *t*(42) < 1, *p =* 0.96, and education level, *t*(42) < 1, *p =* 0.83. Characteristics of blind participants are reported in Table 1. None of the blind participants reported having any prior visual memories. All blind participants were experienced Braille readers, and they were independent travelers.

**Table 1:** Details of the blind participants tested in this study.

Subject	Sex	Age (years)	Highest level of education (years)	Blindness onset	Cause of blindness	Level of visual function
1	Female	46	22	Birth	Retinopathy of prematurity	No light perception in both eyes
2	Male	38	18	Birth	Retinopathy of prematurity	No light perception in both eyes
3	Male	20	13	Birth	Leber’s congenital amaurosis	Light perception in both eyes
4	Male	61	22	Birth	Neonatal glaucoma	Minimal light perception in right eye only; Left: no light perception
5	Male	57	13	1 y/o	Congenital glaucoma	No light perception in both eyes
6	Male	57	13	3 months	Bilateral glaucoma	No light perception in both eyes
7	Male	34	18	Birth	Optic nerve head hypoplasia	No light perception in both eyes
8	Male	60	13	1 year	Buphthalmos	Light perception in left eye only; Right: no light perception
9	Male	58	13	Birth	Unknown	No light perception in both eyes
10	Female	26	13	Birth	Congenital glaucoma, corneal dystrophy	No light perception in both eyes
11	Male	36	16	Birth	Retinopathy of prematurity	No light perception in both eyes
12	Male	38	16	Birth	Retinopathy of prematurity	Minimal light perception in left eye only; Right: no light perception
13	Male	32	18	Birth	Genetic disorder not otherwise specified	Light perception in both eyes
14	Male	45	8	Birth	Rubella during pregnancy	Minimal light perception in left eye only; Right: no light perception
15	Female	26	18	Birth	Peter’s anomaly	No light perception in both eyes
16	Female	31	18	Birth	Leber’s congenital amaurosis	No light perception in both eyes
17	Female	34	18	Birth	Genetic disorder not otherwise specified	No light perception in both eyes
18	Female	55	18	1 y/o	Retinoblastoma	No light perception in both eyes
19	Male	51	16	Birth	Retinopathy of prematurity	Light perception in both eyes
20	Male	60	22	Birth	Retinopathy of prematurity	No light perception in both eyes
21	Male	55	8	Birth	Genetic disorder not otherwise specified	No light perception in both eyes
22	Female	32	18	Birth	Retinopathy	No light perception in both eyes

Informed consent was obtained from all participants. The study was approved by the local ethical committee and was carried out in accordance with the Declaration of Helsinki.

### Stimuli

Stimuli consisted of 40 spontaneous vocalizations (20 positive, 20 negative) identical to those used in the study of Atias et al. (2019). Stimuli were brief vocalization “bursts” that portrayed the first reaction of a vocalizer involved in real-life situations of intense joy or intense fear. Examples of the scenarios for fearful and joyful situations were pranks to believe a snake or a spider is in one’s home and the announcement of a new baby to grandparents, respectively. For each scenario, vocalizations were selected if they did not include any verbal information, were expressed by a single expresser, and if no auditory cue other than the expresser’s vocalization was present in the recording [see Supplementary Material of Atias et al. (2019) for additional information on the selection and editing of the vocalizations]. The duration of the vocalizations ranged from 400 to 2,600 ms (mean duration = 876 ms, *SD* = 390 ms; median duration = 847 ms).

### Procedure

The experiment was conducted in person. Participants were seated comfortably and wore headphones. Sighted control participants were blindfolded during the entire experiment. The experiment consisted of an emotional evaluation task similar to that used in the study of Atias et al. (2019). Specifically, participants were instructed to evaluate the valence (“Is the emotion expressed positive or negative?) and the intensity (i.e., “How intense is the emotion expressed?”) of real-life vocalizations. Participants rated the valence and the intensity of the same 40 vocalizations in two different blocks using a 1–9 Likert scale (for the valence block: 1 = *extremely negative*, 9 = *extremely positive*; for the intensity block: 1 = *not intense at all*, 9 = *extremely intense;* presentation order was counterbalanced across participants). At the beginning of each block, the participants were told whether they had to evaluate the valence or the intensity of the presented stimuli. After listening to each vocalization, participants verbally expressed their evaluation, and the experimenter pressed the corresponding number key on the response keyboard. Stimuli presentation and data recording were implemented using E-prime 2.0 software. The experiment lasted approximately 30 min (including instructions and debriefing).

### Statistical analysis

As with the previous study of Atias et al. (2019), valence ratings were transformed (by applying the formula: “X – 5”) such that positive values represent increasingly positive valence judgments (1 to 4) and negative values represent increasingly negative valence judgments (−1 to −4). To evaluate the possible effect of blindness on valence recognition, we performed a mixed repeated-measures ANOVA, with Valence (negative vs. positive) as a within-subjects factor and Group (blind vs. sighted) as a between-subjects factor. A similar ANOVA was performed on Intensity scores.

## Results

**Figure 1:**
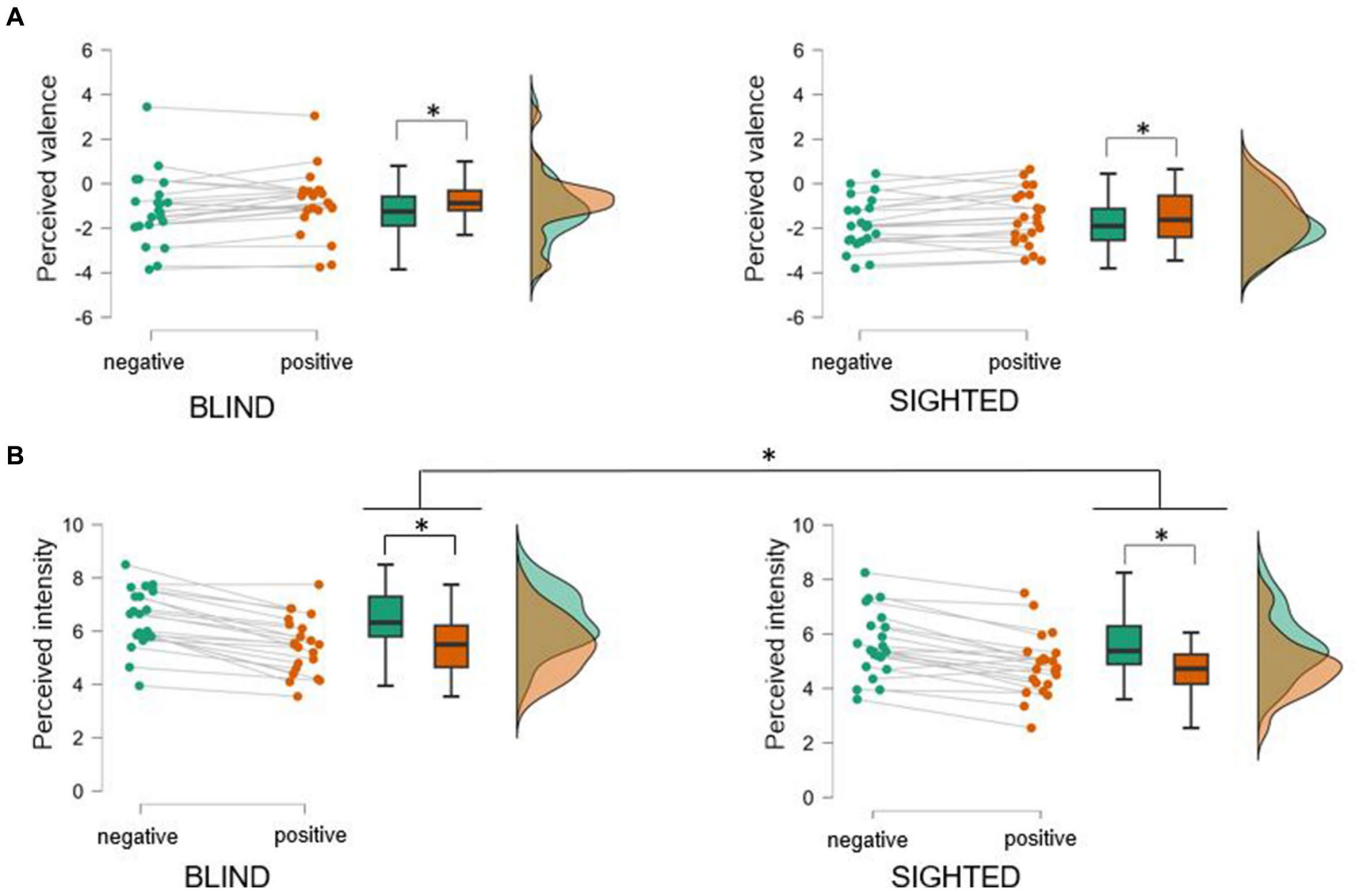
Perceived valence **(A)** and intensity **(B)** of real-life vocalizations evoked in intense positive and negative situations in early blind and sighted participants. Overall, both blind and sighted participants rated all the vocalizations as negative irrespective of their original valence (note that negative vocalizations were perceived as slightly more negative than positive ones). Both groups assigned moderate/high intensity to the vocalizations, with negative vocalizations beeing perceived more intense than positive onse. Critically, blind individuals rated the intensity of all vocalizations as higher compared to sighted controls. Asterisks indicate significant differences (*ps* < 0.05) between experimental conditions.

Mean valence ratings from both positive (*M* = −1.22, *SD* = 1.37) and negative vocalizations (*M* = −1.48, *SD* = 1.41) were found to be in the negative valence range. This suggests that overall, participants tended to interpret all vocalizations as emotionally, negative (see Figure 1A). The mixed repeated-measures ANOVA on valence recognition revealed a significant main effect of Valence, *F*(1,42) = 9.01, *p* = 0.005, η_p_^2^ *= 0*.18, indicating that participants identified positive vocalizations as slightly more positive than negative ones. Neither the main effect of Group, *F*(1,42) = 2.56, *p* = 0.12, nor the interaction Valence by Group, *F*(1,42) = 0.0007, *p* = 0.98, reached statistical significance.

Regarding intensity scores, vocalizations were overall perceived at an intermediary level of intensity (just above the middle score of the intensity 1–9 Likert scale) (see Figure 1B). The mixed repeated-measures ANOVA on intensity scores revealed a main effect of Valence, *F*(1,42) = 103.51, *p* < 0.001, η_p_^2^ *= 0*.71, indicating that negative vocalizations were rated more intense than positive. Furthermore, the factor of Group was significant, *F*(1,42) = 4.86, *p* = 0.033, η_p_^2^ *= 0*.10, indicating that blind participants perceived the vocalizations to be more intense (*M* = 5.94, *SD* = 1.05) than sighted participants (*M* = 4.95, *SD* = 1.14). The interaction Valence by Group, *F*(1,42) = 0.915, *p* = 0.34, was not statistically significant.

## Discussion

The capacity to understand others’ emotions and react accordingly is a key social ability that may be compromised in case of a profound sensory loss. Here we specifically investigated whether early blindness affects the capacity to interpret emotional vocalizations, whose valence may be difficult to recognize in the absence of a meaningful context (e.g., Atias et al., 2019). We found that blind and sighted participants found discriminating the valence of intense emotional vocalizations similarly difficult when no other contextual information was provided. Nonetheless, blind participants rated the affective vocalizations as more intense than sighted controls, possibly reflecting a higher perceived saliency.

The intensity of non-verbal vocalizations is characterized by specific acoustic profiles. Intense vocal stimuli are associated with increased duration, high fundamental frequency (pitch), and more high-frequency energy in the spectrum (bright timbre) (Anikin, 2020). These features inform about the significance and saliency of the stimuli, therefore attracting (automatic/bottom-up) attention and facilitating information prioritization and efficiency of appraisal (Belin and Zatorre, 2015; Anikin, 2020). For instance, screams have acoustic features dedicated to alarm signals but do not inform about the specific content meaning (fear vs. anger) of the emotion conveyed or the speaker’s identity (Anikin, 2020). Our findings indicate that blind participants are more sensitive than sighted individuals to these intensity-related acoustic characteristics, possibly because they represent a unique or primary source of sensory information during social interactions signaling danger or possible negative outcomes. Similar evidence was previously reported by Klinge et al. (2010, 2012) also showing that blindness is associated with greater amygdala sensitivity in response to emotional prosodic stimuli (and in particular negative ones) suggesting a neural mechanism for this observed behavioral compensation (Klinge et al., 2010, 2012). Interestingly, this finding may also be interpreted considering the higher interoceptive capacity observed in blind individuals (Radziun et al., 2023). High interoceptive ability is associated with increased sensitivity to the emotions of others (Terasawa et al., 2014) and with increased connectivity of the salience network (Chong et al., 2017). This may contribute to the perceived higher intensity of emotional stimuli by blind individuals, a hypothesis that deserves further investigation.

Our data show that blind participants performed similarly to the sighted controls in processing the valence of spontaneous intense vocal expressions. In particular, and regardless of visual status, participants rated both positive and negative vocalizations in the negative range of the valence scale, thus not reliably differentiating their valence, and replicating the previous findings of Atias et al. (2019). Hence, blindness does not seem to lead to an advantage in emotional recognition of vocal stimuli [see Gamond et al. (2017), Martins et al. (2019), Chen et al. (2022), and Sarzedas et al. (2024)], as with other domains such as spatial auditory localization or sentence comprehension (Battal et al., 2020; Loiotile et al., 2020). One possibility is that blind individuals do not develop better competencies in this domain because they may rely on other sources of contextual information such as verbal content [see Occelli et al. (2017)]. Indeed, in social interactions in everyday life, vocal emotional cues are usually accompanied by verbal semantic information. This may be more common for blind individuals who often rely on what other people say or on audio descriptions when accessing certain forms of media (e.g., television). Moreover, prior evidence also suggests that blindness may not affect social cognition abilities overall, which may develop via other sources [Ricciardi et al., 2009; for a recent meta-analysis, see Arioli et al. (2021)]. For instance, blind and sighted individuals similarly evaluate others’ trustworthiness based on the pitch of the voice (Oleszkiewicz et al., 2017) and similarly form social impressions of others [Ferrari et al., 2017; Arioli et al., 2024; see Bedny et al. (2009) and Arioli et al. (2021), for evidence on similar neural correlates of social cognition in the blind and sighted]. Overall, this suggests that blindness-related compensatory mechanisms in the auditory domain are restricted to specific auditory functions such as navigation/orientation, as well as spatial and language processing (Cattaneo et al., 2008, 2010b; Handjaras et al., 2016; Battal et al., 2020; Loiotile et al., 2020), rather than being generalized to the socioemotional domain. The advantage of blind individuals in language processing might be sufficient to ensure proper emotional and social competence or at least, socioemotional skills that are comparable to that of sighted people.

In interpreting our findings, some limitations should be acknowledged. The first limitation relates to the stimuli we used. Indeed, to make the task more sensitive to detect possible group differences, we intentionally employed ambiguous intense vocalizations (Atias et al., 2019) without providing any contextual information. The task may have been therefore too demanding hiding possible group differences. Prior evidence suggests that emotional stimuli may influence behavior only when their emotional content is relevant to the participants’ goal given the context in which they operate (e.g., Calbi et al., 2022; Mancini et al., 2022; Mirabella et al., 2023). Accordingly, future studies may test whether “contextualized” vocalizations elicit different reactions in blind and sighted participants, possibly also employing more nuanced vocalizations [see Cowen et al. (2019)] as well as physiological indexes of emotional arousal (e.g., skin conductance response, heart rate). A second limitation relates to the lack of control tasks in our study. In particular, one may object that the higher intensity rates reported by blind participants may represent an unspecific effect possibly extending to other auditory (non-emotional) stimuli or reflecting response biases, and this possibility should be controlled for in an additional task. However, in our study we specifically asked participants to evaluate the intensity of the emotion expressed and not “loudness” of the sounds, and prior studies have shown that early blind individuals exhibit similar sensitivity thresholds for (neutral) sounds’ intensity discrimination [Voss and Zatorre, 2012; for a review see Sabourin et al. (2022)].

In summary, although our findings are preliminary and deserve further investigation, our study represents the first attempt to explore the effect of early blindness on the perception of spontaneous emotional vocalizations.

## Data availability statement

The dataset for this article is not publicly available due to concerns regarding participants’ anonymity. Requests to access the datasets should be directed to the corresponding author.

## Ethics statement

The studies involving humans were approved by Research Integrity and Ethics Committee of the University of Bergamo. The studies were conducted in accordance with the local legislation and institutional requirements. The participants provided their written informed consent to participate in this study.

## Author contributions

CF: Conceptualization, Data curation, Formal analysis, Investigation, Methodology, Project administration, Software, Visualization, Writing – original draft, Writing – review & editing. MA: Data curation, Formal analysis, Investigation, Methodology, Project administration, Visualization, Writing – original draft, Writing – review & editing. DA: Methodology, Writing – review & editing. LM: Supervision, Validation, Writing – review & editing. ZC: Conceptualization, Formal analysis, Funding acquisition, Investigation, Methodology, Resources, Supervision, Validation, Visualization, Writing – original draft, Writing – review & editing.
